# Supplementary material for: Comparison of kinesiotape, counterforce brace, and corticosteroid injection in patients with tennis elbow: A prospective, randomized, controlled study
Source: PLoS One. 2025 Jul 18;20(7):e0328396. doi: 10.1371/journal.pone.0328396 (PMC12273928; doi:10.1371/journal.pone.0328396)
Supplement: S1 File — (PDF) [file pone.0328396.s001.PDF]

بسمه تعالی

دانشگاه علوم پزشکی بابل

معاونت تحقیقات و فناوری
طرح پیشنهادی تحقیق

مشخصات طرح پژوهشی

نوع پایان نامه/طرح ارائه شده	پایان نامه
آیا این طرح یا پایان نامه محصول محور است؟	☐
زمینه کاربردی طرح یا پایان نامه	
نحوه مشارکت طرح	
محل اجرای طرح	بیمارستان شهید بهشتی بابل

عنوان طرح	مقایسه اثرات کنزیوتیپ، کانترفورس بریس و تزریق کورتیکواستروئید بر ضخامت تاندون اکستنسور مشترک انگشتان و شدت درد در بیماران مبتلا به تنیس البو
عنوان لاتین طرح	Comparing the effects of kinesio taping, counterforce brace and corticosteroid injection on common extensor tendon thickness and pain intensity in patients with tennis elbow.
سازمان متبوع	مرکز تحقیقات اختلال حرکت(پژوهشکده سلامت) دانشگاه علوم پزشكي بابل
زمان اجرای طرح -ماه	12
آیا طرح پایان‌نامه دانشجویی است؟	☑
نوع پایان نامه	كارشناسي ارشد
آیا دانشجو شهریه پرداز می باشد؟	☐
دانشکده	توانبخشی
گروه	فیزیوتراپی
حیطه اولویت مرتبط	بيماريهاي غيرواگير
اولویت های دیگر	
اولویت مرتبط	اپيدميولوژي، پيشگيري، درمان و بازتواني اختلالات اسكلتي- عضلاني
خلاصه پروپوزال	اپی کندیلیت خارجی شایع ترین علت درد در قسمت خارجی آرنج بوده، که همچنین به عارضه آرنج تنیس بازان و یا بیماری تنیس البو شناخته می شود. استفاده از روش های تهاجمی مانند تزریق کورتیکو استروئید در محل اتصال تاندون های اکستنسور مچ دربین پزشکان شایع است. از روش های غیر تهاجمی که برای کنترل و بهبود درد این بیماران به کار می رود می توان به کانترفورس بریس و کنزیوتیپ اشاره کرد. با توجه به آنکه بیماری تنیس البو هزینه های مالی زیادی را بر جامعه، به خصوص جمعیت بزرگسال تحمیل کرده است و فرد را از

حضور در کارهای شغلی و اجتماعی باز می دارد بررسی مقایسه اثرات درمان های تهاجمی مانند تزریق کورتیکو استروئید و درمان های غیر تهاجمی مانند کینزیوتیپ و کانترفورس بریس، حائز اهمیت می باشد. در این مطالعه بیماران در سه گروه 15 نفره تقسیم خواهند شد. گروه اول کینزیوتیپ، گروه دوم کانترفورس بریس و گروه سوم کورتیکو استروئید دریافت می کنند. معیارهای اولیه اندازه گیری شامل ضخامت تاندون اکستنسور مشترک انگشتان و شدت درد می باشد. معیارهای ثانویه شامل قدرت گریپ و ناتوانی عملکردی اندام فوقانی می باشد. معیارهای اندازه گیری قبل از درمان، بعد از درمان و دوهفته بعد از درمان اندازه گیری می شوند.در انتهای مطالعه بعد از جمع آوری داده ها ابتداً به منظور بررسی توزیع داده ها از آزمون kolmogorov smirnov test استفاده خواهد شد. بر حسب نتایج این آزمون از آزمون های پارامتری (independent sample test,paired sampels T-test, one way ANOVA) ویا آزمون های غیرپارامتری (Mann-whitney U test, Wilcoxon singed rank test,chi-squared test)استفاده خواهد شد.

نوع مطالعه	کارآزمايي باليني
نوع طرح	کاربردی
رسته	misc
رسته/ زیررسته	

مشخصات مجری/همکاران طرح

نام و نام خانوادگی	سمت در طرح	نوع همکاری	دوره تحصیلی	رشته تخصصی	مرتبه علمی	پست الکترو
سید محمدرضا حسینی(8963)	مجری	دانشجو	کارشناس ارشد	فیزیوتراپی	دانشجو	mail.com
محمد تقی پور درزی(49)	مجری مسئول	استاد راهنما اول	دکتری تخصصی	فیزیوتراپی	دانشیار	ahoo.com
رحمت اله جوکار(4926)	مجری	استاد راهنما دوم	دکتری تخصصی پزشکی	ارتوپدی	استادیار	mail.com
مهرداد مصطفی لو(5672)	همکار اصلی	استاد مشاور	دکتری تخصصی پزشکی	رادیولوژی	استادیار	ahoo.com
هدی شیرافکن(5150)	همکار اصلی	استاد مشاور	دکتری تخصصی	آمار زیستی	استادیار	mail.com
خدابخش جوانشیر(61)	همکار اصلی	مشاور طرح	دکتری تخصصی	فیزیوتراپی	دانشیار	ahoo.com

کلیات و مقدمه

عنوان	متن
مقدمه و بیان مساله	مساله تحقیق:<br><br>اپی کندیلیت خارجی شایع ترین علت درد در قسمت خارجی آرنج بوده، که همچنین به عارضه آرنج تنیس بازان و یا بیماری تنیس البو[1] شناخته می شود. این بیماری اغلب به عنوان یک عارضه مرتبط با کار و فعالیت بوده و بنابراین یک مساله سلامت جهانی می باشد. با توجه به آنکه سالانه حدود 1 تا 3 درصد جمعیت بزرگسالان را در سراسر جهان تحت تاثیر قرار می دهد، پیشگیری، کنترل درد ناشی از این بیماری و درمان آن از اهمیت ویژه ای برخوردار است. استفاده از روش های تهاجمی مانند تزریق کورتیکو استرویید در محل اتصال تاندون های اکستنسور مچ دربین پزشکان شایع است. از روش های غیر تهاجمی که برای کنترل و بهبود درد این بیماران به کار می رود می توان به کانترفورس بریس[2] و کینزیوتیپ[3] اشاره کرد. با توجه به آنکه بیماری تنیس البو

عنوان	متن
	هزینه های مالی زیادی را بر جامعه، به خصوص جمعیت بزرگسال تحمیل کرده است و فرد را از حضور در کارهای شغلی و اجتماعی باز می دارد، بررسی مقایسه اثرات درمان های تهاجمی مانند تزریق کورتیکو استروویید و درمان های غیر تهاجمی مانند کینزیوتیپ و کانترفورس بریس، حائز اهمیت می باشد.<br><br>مقدمه و بیان مساله:<br><br>اپی کندیلیت خارجی که به طور معمول تحت عنوان عارضه آرنج تنیس بازان و یا بیماری تنیس البو شناخته می شود که از شایع ترین دلیل درد آرنج می باشد که بر اساس گزارش ها سالانه حدود 1 تا 3 درصد از جمعیت بزرگسالان را تحت تاثیر قرار می دهد. این بیماری به طور اولیه در اثر التهاب های ثانویه یا به طور مستقیم در اثر جراحت تروماتیک و استفاده مکرر و بیش از حد از عضلات اکستنسور مچ که به اپی کندیل خارجی استخوان بازو متصل می شوند، ایجاد می گردد (1). این آسیب با تغییرات تدریجی بافت تاندونی همراه می‌باشد و معمولا به عنوان التهاب تاندون اکستانسور کارپی رادیالیس برویس تعریف می شود (2, 3). اما در 50-35 درصد موارد، عضله اکستنسور مشترک انگشتان را هم می‌تواند درگیر کند(4, 5). ضایعات ممکن است براساس شدت و میزان پیشرفت بیماری، انتزیس (محلی که رباط‌ها و تاندون‌ها به استخوان متصل می‌شوند) یا تاندون ها را تحت تاثیر قرار دهد و حتی در نهایت می تواند سبب ایجاد درد در قسمت قدام عضلات مربوطه و ضعف و نکروز فیبرهای آن عضلات گردد (6). کشش مکرر بیش از حد مجموعه عضله-تاندون یک فاکتور پاتوفیزیولوژی برای ایجاد این ضایعات و پیشرفت به سمت مراحل مزمن بیماری می باشد (1). بیماری تنیس البو یک عارضه تخریبی می باشد که با افزایش ضخامت در تاندون اکستنسور مشترک و تداخل در عملکرد مچ به خصوص در هنگام مشت کردن تشخیص داده می شود (7). البته حین ارزیابی باید به ناحیه گردن، عصب رادیال و ناحیه ستون فقرات سینه نیز توجه کرد .به خصوص زمانیکه این اختلالات همراه با هم وجود دارند(درد گردن یا درد بازو یا گزگز و پاراستزی) (8). یافته های اولتراسونوگرافی می تواند در تعیین مقدار ضخامت تاندون، کلسیفیکاسیون، بی نظمی استخوانی، رسوب کلسیفیه و تغییرات دژنراتیو تاندونی کمک کننده باشد (9, 10). درمان بیماری تنیس البو معمولاً با پنج هدف انجام می شود: 1. کنترل درد آرنج 2. کنترل حرکت اندام درگیر 3. بهبود قدرت گریپ 4. بازگشت عملکرد نرمال اندام درگیر 5. جلوگیری از پیشرفت عارضه. روش های درمانی غیرتهاجمی در بیماری تنیس البو

متن	عنوان
شامل داروهای ضد التهابی، فیزیوتراپی و همچنین استفاده از یخ در مرحله التهابی بیماری می باشد (11, 12). از روش های درمانی تهاجمی می توان تزریق خون غنی از پلاسما[4] و تزریق کورتیکو استرویید را نام برد. (13, 14). از رایج ترین روش های درمانی تهاجمی، تزریق موضعی کورتیکواسترویید به همراه بی حس کننده های موضعی می باشد (15). اگرچه مطالعاتی برتری تزریق کورتیکو استرویید را نسبت به سایر درمان های محافظه کارانه گزارش دادند، اما مطالعاتی هم عوارض جانبی مانند اکیموز، استئومیلیت، آتروفی بافت چربی زیر جلدی و هیپوپیگمانتاسیون را پس از تزریق آن نشان دادند (16). درمان های مختلف فیزیوتراپی یکی از استراتژی های امیدبخش برای درمان بیماری تنیس البو می باشد (17).از روش های درمانی رایج بیماری تنیس البو، کانترفورس بریس می باشد. تصور می شود کانترفورس بریس با ایجاد مبدا مصنوعی برای تاندون عضلات اکستنسور مشترک انگشتان می تواند میزان استرس وارد بر تاندون را کاهش دهد (18). یکی دیگر از روش های فیزیوتراپی برای درمان بیماری تنیس البو، کینزیوتیپ می باشد که در دهه گذشته در بین ورزشکاران و بیماران برای جلوگیری از آسیب، تقویت عملکرد و توانبخشی رایج شده است. چندین مطالعه نشان می دهد که کینزیوتیپ از طریق تسهیل یا مهار فعالیت عضله سبب کاهش علائم بیماری می شود (19). بررسی مطالعات قبلی نشان می دهد عدم اجماع در مورد اثربخشی درمان‌های غیرتهاجمی و تهاجمی و همچنین پیش‌آگهی‌های مختلف بیماران، روند درمان را با مشکل مواجه می‌کند. چندین مطالعه درمورد مقایسه درمان های غیر تهاجمی هم انجام شده است، ولی هیچ یک از آنها برتری خود را نسبت به سایرین نشان نداده اند. تا آنجاییکه ما اطلاع داریم، مطالعه ای در رابطه با مقایسه اثربخشی سه روش کینزیوتیپ، کانترفورس بریس و تزریق کورتیکو استرویید در بهبودی بیماران تنیس البو انجام نشده است. هم چنین بیشتر مطالعات با پیامدهای سابجکتیو یا عینی به بررسی معیارهای اندازه گیری قبل و بعد از درمان پرداخته اند و مطالعات کمی به صورت متمرکز تغییرات ضخامت تاندون در ناحیه اپی کندیل خارجی را با استفاده از یافته های دستگاه اولتراسونوگرافی بعد از انجام روش های درمانی، در بیماران تنیس البو مورد تجزیه و تحلیل قرار دادند. لذا هدف از مطالعه حاضر، بررسی اثرات این سه روش در تغییرات میزان درد، عملکرد اندام فوقانی، قدرت گریپ[5] و همچنین با استفاده از دستگاه سونوگرافی، میزان تغییرات ضخامت تاندون اکستنسور مشترک انگشتان[6] در بیماران مبتلا به عارضه تنیس البو می باشد.<br><br>[1] Tennis elbow<br><br>[2] Counterforce brace<br><br>[3] Kinesio tape<br><br>[4] Platelet-rich plasma (*PRP*)<br><br>[5] grip strength	

عنوان	متن
	Common Extensor Tendon (CET) [6]
بررسی متون	**مقالات کانترفورس بریس در تنیس البو**<br><br>1.بیست(Bisset) و همکارانش در سال 2014 مطالعه ای را به صورت RCT بر روی 34 بیمار تنیس البو به منظور بررسی کارایی فوری کانترفورس بریس کانترفورس بریس به همراه تعلیق بند آرنج انجام دادند. همه افراد هر دو نوع بریس را دریافت کردند. همچنین معیارهای اندازه گیری در حالت بدون بریس هم اندازه گیری شد. نتایج مطالعه آنها نشان داد که قدرت گریپ و آستانه تحمل درد[1] به طور معناداری در حین استفاده از دو بریس بهبود پیدا کرد. با این وجود سایر فاکتورها همچون زاویه مچ در هنگام گریپ بین سه گروه مطالعه تفاوت معناداری نشان نداد (18).<br><br>2. براتی(Barati)و همکارانش در سال 2018 مطالعه ای را به صورت RCT بر روی 50 بیمار تنیس البو به منظور بررسی اثرات حسی-حرکتی فوری بریس انجام دادند. بیماران هر دو نوع کانترفورس بریس و کانترفورس بریس به همراه تعلیق بند آرنج را دریافت کردند. نتایج مطالعه آنها نشان داد که شدت درد، قدرت گریپ ،مهارت حرکات ظریف دست و حس عمقی آرنج بیماران در هنگام استفاده از کانترفورس بریس و کانترفورس بریس به همراه تعلیق بند آرنج نسبت به وضعیت بدون بریس به طور معناداری بهبود یافت. همچنین نتایج مطالعه آنها نشان داد تفاوت معناداری بین دو روش کانترفورس بریس و کانترفورس بریس به همراه تعلیق بند آرنج در معیارهای اندازه گیری مطالعه مشاهده نشد. (20)<br><br>3. در سال 2015، داندر(Dundar) و همکارانش مطالعه ای را به صورت RCT جهت بررسی مقایسه اثرات لیزر پرتوان و کانترفورس بریس بر روی 91 بیمار مبتلا به تنیس البو انجام دادند. بیماران در سه گروه درمان با لیزر پرتوان، کانترفورس بریس و گروه کنترل که لیزر پلاسبو دریافت کردند، تقسیم شدند. بیماران در گروه لیزر پرتوان، به مدت 15 روز در طول 3 هفته لیزر دریافت کردند. در گروه دیگر، بیماران به مدت 4 هفته از کانترفورس بریس استفاده کردند. معیارهای اندازه گیری شامل قدرت گریپ، شدت درد، ناتوانی عملکردی و همچنین ارزیابی ضخامت تاندون اکستنسور مشترک انگشتان به وسیله دستگاه اولتراسونوگرافی بود. نتایج حاصل از مطالعه آنها نشان داد که در دو گروه کانترفورس بریس و لیزر پرتوان شدت درد، قدرت گریپ و امتیاز ناتوانی عملکردی نسبت به گروه کنترل به طور معناداری بهبود یافت. اگرچه یافته های اولتراسونوگرافی تفاوت معناداری بین گروه های درمان و گروه کنترل نشان ندادند (21).<br><br>4. اکن(Oken) و همکارانش در سال 2008، مطالعه ای را به صورت RCT جهت مقایسه اثرات کوتاه مدت کانترفورس بریس، لیزر کم توان و اولتراسوند بر روی 59 بیمار به بیماری تنیس البو انجام دادند. برنامه تمرین درمانی به صورت روتین به تمام بیماران داده شد. در گروه کانترفورس بریس(20نفر)، بیماران به مدت 2هفته بریس

عنوان	متن
	دریافت کردند. در گروه لیزر(20نفر) و گروه اولتراسوند(19نفر)، بیماران هات پک هم دریافت کردند. بیماران در گروه لیزر، 10 جلسه درمانی به مدت 2هفته لیزر و در گروه اولتراسوند، 10 جلسه درمانی به مدت 2هفته اولتراسوند دریافت کردند. قدرت گریپ و شدت درد، قبل از شروع درمان، دو هفته و شش هفته پس از درمان اندازه گیری شدند. نتایج حاصل از مطالعه آنها نشان داد شدت درد در هر سه گروه به طور معناداری بهبود پیدا کرد ولی قدرت گریپ تنها در گروه لیزر کم توان بهبود یافت (22).<br><br>**مقالات کینزیوتیپ و کورتیکواسترویید در تنیس البو**<br><br>1. در سال 2019، کوکاک(Koçak) و همکارانش مطالعه ای را به صورت RCT به منظور بررسی تاثیر کوتاه مدت تزریق استرویید در مقایسه با چسباندن کینزیوتیپ بر روی شدت درد، قدرت گریپ بدون درد و همچنین ناتوانی عملکردی در بیماران مبتلا به تنیس البو انجام دادند. بیماران به صورت تصادفی در سه گروه کینزیوتیپ(28 نفر)، گروه استرویید(28 نفر) و گروه کینزیوتیپ به همراه تزریق استرویید(28 نفر) تقسیم بندی شدند. در گروه کینزیوتیپ و کینزیوتیپ به همراه تزریق استرویید، بیماران دوبار در هفته به تعداد پنج جلسه کینزیوتیپ دریافت کردند. نتایج مطالعه آنها نشان داد در گروه کینزیوتیپ و گروه تزریق استرویید، تنها قدرت گریپ در انتهای هفته دوازدهم به طور معناداری بهبود یافته بود. درحالی که در گروه کینزیوتیپ به همراه تزریق استرویید تقریبا در تمام معیارهای اندازه گیری شاهد تفاوت معنادار در آنالیزهای آماری نسبت به دو گروه دیگر بودند (23).<br><br>2. ازمن(Ozmen) و همکارانش در سال 2021، جهت بررسی مقایسه اثرات کینزیوتیپ، شاک ویو و اولتراسوند برروی ضخامت تاندون عضله اکستنسور مشترک انگشتان، شدت درد، قدرت گریپ و همچنین امتیاز PRTEE[2]، مطالعه ای را به صورت RCT در بیماران مبتلا به تنیس البو انجام دادند. بیماران در سه گروه کینزیوتیپ(13نفر)، شاک ویو(14) و گروه اولتراسوند(13 نفر)، تقسیم بندی شدند. در گروه کینزیوتیپ، هر دو روز یک بار به مدت دو هفته، تیپ ها عوض می شدند. برنامه روتین فیزیوتراپی شامل گذاشتن هات پک و تحریک الکتریکی به هر سه گروه داده شد. نتایج حاصل از مطالعه آنها نشان داد شدت درد در هر سه گروه به طور معناداری کاهش یافت. قدرت گریپ پس از 8 هفته تنها در گروه کینزیوتیپ افزایش یافت. امتیازPRTEE پس از دو هفته در دو گروه اولتراسوند و شاک ویو و پس از گذشت هشت هفته در هر سه گروه به طور معناداری بهبود پیدا کرد. ضخامت تاندون عضله اکستنسور مشترک انگشتان هم پس از هشت هفته تنها در گروه شاک ویو به طور معناداری کاهش پیدا کرده بود (24).<br><br>3. در سال 2019، مطالعه ای به صورت RCT به منظور بررسی اثر کینزیوتیپ بر روی 30 بیمار تنیس البو توسط گری(Giray) و همکارانش انجام شد. بیماران به صورت تصادفی در سه گروه شامل کینزیوتیپ به همراه تمرین، تیپ پلاسبو به همراه تمرین و گروه کنترل که فقط برنامه تمرین درمانی انجام می دادند، تقسیم بندی شدند. برنامه تمرین درمانی شامل تمرینات قدرتی و استرچینگ بود. در گروه های کینزیوتیپ و تیپ

متن	عنوان
پلاسبو، تیپ ها هر 3الی 4 روز به مدت دو هفته عوض می شدند. ارزیابی بیماران شامل [3]VAS ، امتیاز PRTEE و قدرت گریپ بود که قبل، پس از درمان و 4 هفته پس از درمان انجام شد. نتایج حاصل از مطالعه آنها نشان داد که امتیاز PRTEE بعد از انجام درمان و در پیگیری 4 هفته ای پس از درمان در گروه کینزیوتیپ به طور معناداری نسبت به دو گروه دیگر بهبود یافته بود. همچنین کینزیوتیپ باعث افزایش قدرت گریپ و کاهش نمره VAS در حالت استراحت شد (25).<br><br>4. اراسلان(Eraslan) و همکارانش در سال 2017، مطالعه ای را به صورت RCT به منظور بررسی اثرات درمانی کینزیوتیپ در مقایسه با شاک ویو بر روی 45 بیمار تنیس البو انجام دادند. بیماران به صورت تصادفی در سه گروه تقسیم بندی شدند. همه بیماران برنامه روتین فیزیوتراپی شامل یخ، تحریک الکتریکی و همچنین برنامه تمرین درمانی در منزل را دریافت کردند. بیماران در گروه کینزیوتیپ، 5 روز در هفته به مدت 3هفته کینزیوتیپ دریافت کردند. نتایج حاصل از مطالعه آن ها نشان داد که شدت درد و قدرت گریپ بدون درد در گروه کینزیوتیپ در مقایسه با گروه شاک ویو و گروه کنترل به طور معناداری بهبود پیدا کردند (26).<br><br>5. ارپالا(Erpala) و همکارانش در سال 2021، مطالعه ای را به صورت RCT جهت بررسی اثرات فوری کینزیوتیپ و تزریق کورتیکو استرویید بر روی 50 بیمار مبتلا به تنیس البو انجام دادند. بیماران در سه گروه کینزیوتیپ(20نفر)، گروه استرویید(15نفر) و گروه استراحت و دارو(15نفر) تقسیم بندی شدند. در گروه کینزیوتیپ، بر روی بیماران 3 بار تیپ به فاصله زمانی 5 روز زده شد. نتایج حاصل از مطالعه آنها نشان داد همه معیارهای اندازه گیری شامل امتیاز Nirschl، شدت درد، ناتوانی عملکردی اندام فوقانی و همچنین معیار(PRTEE) در هر سه گروه در پایان هفته دوم وهفته چهارم بهبود یافت. اما تنها در گروه کینزوتیپ شاهد تفاوت معنادار در انتهای هفته چهارم در معیارهای اندازه گیری بودند (16).<br><br>6. مطالعه ای در سال 2017 به صورت به صورت RCT توسط هسیه(Hsieh) و همکارانش به منظور مقایسه بین تزریق کورتیکو استرویید و لیدوکایین در 61 بیمار مبتلا به تنیس البو انجام شد. گروه اول ده میلی گرم تریامسینولون و گروه دوم ده میلی گرم لیدوکایین یک درصد دریافت کردند. نتایج مطالعه آنها نشان داد پس از گذشت دو هفته و دوماه، تفاوت معناداری بین دو گروه در معیار (PRTEE)، شدت درد، ناتوانی عملکردی اندام فوقانی و همچنین قدرت گریپ بدون درد یافت نشد (27).<br><br>[1] Pain threshold	

عنوان	متن
	Patient-Rated Tennis Elbow Evaluation Scale [2]<br><br>Visual Analogue Scale [3]

اهداف و فرضیات

عنوان	متن
هدف اصلی	مقایسه اثرات کینزیوتیپ، کانترفورس بریس و تزریق کورتیکواستروویید بر ضخامت تاندون اکستنسور مشترک، شدت درد، قدرت گریپ و عملکرد اندام فوقانی در بیماران مبتلا به تنیس البو
هدف فرعی	1. مقایسه اثرات کینزیوتیپ، کانترفورس بریس و تزریق کورتیکو استروویید بر قدرت گریپ در بیماران تنیس البو<br><br>2. مقایسه اثرات کینزیوتیپ، کانترفورس بریس و تزریق کورتیکو استروویید بر شدت درد آرنج در بیماران تنیس البو<br><br>3. مقایسه اثرات کینزیوتیپ، کانترفورس بریس و تزریق کورتیکو استروویید بر عملکرد اندام فوقانی در بیماران تنیس البو<br><br>4. مقایسه اثرات کینزیوتیپ، کانترفورس بریس و تزریق کورتیکو استروویید بر ضخامت تاندون اکستنسور مشترک در بیماران تنیس البو
هدف کاربردی	هدف از این مطالعه، ارتقاء سطح کیفیت زندگی و سلامت این بیماران میباشد. نتایج حاصل از این مطالعه می تواند در تغییر دادن رویکرد درمانی بیماران تنیس البو استفاده شود.

عنوان	متن
فرضیه	1. تاثیر کینزیوتیپ، کانترفورس بریس و تزریق کورتیکو استرویید در کاهش درد آرنج در بیماران تنیس البو مشابه است.<br><br>2. تاثیر کینزیوتیپ، کانترفورس بریس و تزریق کورتیکو استرویید بر روی ضخامت تاندون اکستنسور مشترک در بیماران تنیس البو مشابه است.<br><br>3. تاثیر کینزیوتیپ، کانترفورس بریس و تزریق کورتیکو استرویید در افزایش قدرت گریپ در بیماران تنیس البو مشابه است.<br><br>4. تاثیر کینزیوتیپ، کانترفورس بریس و تزریق کورتیکو استرویید در میزان توانایی عملکردی اندام فوقانی در بیماران تنیس البو مشابه است.
سوال	-

جدول متغیرها

نام متغیر	نوع متغیر	مقیاس متغیر	نقش متغیر	تعریف کاربردی	واحد اندازه گیری
سن	كمي پيوسته	فاصله اي	ساير		سال
جنس	كيفي	اسمي	ساير		مرد/زن
BMI	كمي پيوسته	فاصله اي	ساير		kg/m2
قد	كمي پيوسته	فاصله اي	ساير		سانتي متر

نام متغير	نوع متغير	مقياس متغير	نقش متغير	تعريف كاربردی	واحد اندازه گيری
شدت درد	كمي گسسته	فاصله اي	وابسته		VAS
ضخامت تاندون	كمي پيوسته	فاصله اي	وابسته		سانتي متر
قدرت گريپ	كمي پيوسته	فاصله اي	وابسته		نيوتن
عملكرد اندام فوقاني	كيفي	رتبه اي	وابسته		پرسشنامه
تزريق كورتيكو استروئيد	كيفي	نسبتي	مستقل		سي سي
كانترفور بريس	كيفي	نسبتي	مستقل		.
كينزيوتيپ	كيفي	نسبتي	مستقل		-

روش كار

عنوان	متن
جامعه مورد مطالعه/نحوه نمونه گيری و	**شرايط ورود و خروج نمونه‌ها**<br>معيار ورود: (28,27,25)

عنوان	متن
فرمول تعیین حجم نمونه	

1. تندرنس و درد در محل اپی کندیل خارجی
2. مثبت شدن تست‌های تخصصی(Maudsley's test/ Mill's test/ Cozen's test)
3. سن بالای 18 سال
4. داشتن نشانه های بیماری به مدت حداقل 4 هفته تا 6 ماه(بیماران با شدت خفیف تا متوسط

معیار خروج:(3, 25, 28)

1. دردهای رادیکولوپاتی و اسپوندیلولیزیس گردن
2. دیابت
3. پلی نروپاتی و یا نروپاتی های گیر افتادگی
4. سابقه جراحی یا تروما حاد به آرنج
5. بیماری های آرتریت روماتوئید
6. سابقه تزریق استرویید و یا فیزیوتراپی ناحیه لترال اپی کندیل هومروس
7. حاد بودن عارضه(تندرنس شدید، داشتن درد درحرکات اکتیو معمولی، گرم بودن موضع اپی ک
8. سابقه جراحی و یا در رفتگی آرنج
9. بارداری
10. سرطان

***حجم نمونه:**

حجم نمونه با استفاده از نرم افزار G*POWER برای محاسبهRepeated measure ANOVA

Within-between interaction بدست آمد. با توجه به نتایج مقالات(22, 29) مقدار اندا
برابر0.15 در نظر گرفته شد. با فرض خطای 0.05 و توان 80 درصد برای 3 بار ارزیابی در شروع
پس از شروع مطالعه(پایان درمان) و چهار هفته پس از شروع مطالعه و فرض همبستگی 0.6 بی
فرد، حداقل تعداد نمونه مورد نیاز در هر گروه برابر15 نفر بدست آمد. با احتساب 10درص
مجموع51 نفر وارد مطالعه خواهند شد(هر گروه 17 نفر).

روش تصادفی سازی و مخفی سازی روند درمان:

تصادفی سازی با روش Permuted block randomization انجام خواهد شد. اندازه هر بلوک
می شود. در هر بلوک به تعداد مساوی از هر گروه وجود دارد. به علاوه ترتیب قرار گرفتن سه د
تصادفی می باشد و توسط متخصص آمار مشخص می گردد. در پاکت های نامه حروف الف، ب
شوند و بیماران به صورت تصادفی یک پاکت نامه را بر می دارند و بر اساس آن گروه درمانی
شود. بیمارانی که پاکت حرف الف را بردارند در گروه درمانی کانترفورس بریس، بیمارانی که پ
بردارند در گروه درمانی کینزیوتیپ و بیمارانی که پاکت پ را بردارند در گروه تزریق کورتیکو ا

عنوان	متن
	گیرند. بر روی هر پاکت یک کد تصادفی نوشته می شود و با ورود افراد به مطالعه یک پاکت (با تر اختصاص می یابد. کد روی پاکت بر روی پرونده (چک لیست)بیمار نوشته خواهد شد.

کورسازی: در این مطالعه که به صورت کارآزمایی تصادفی کنترل شده یا RCT انجام خواهد شد، ف و درمانگر، دو فرد متفاوت می باشند.لذا این مطالعه از نوع تک سویه کور می باشد. همچنین تحل گروه بندی بی اطلاع است.

انجام معاینات بالینی و تستهای اختصاصی محرک درد در این ناحیه که این تستها شامل:

1. **Maudsley’s test**: بیمار می نشیند و آرنج را ۹۰ درجه و ساعد را در وضعیت پروناسیو اکستانسیون انگشت میانی را در برابر مقاومت ما انجام می دهد. درد در ناحیه خارجی آرنج ن تست است (30).

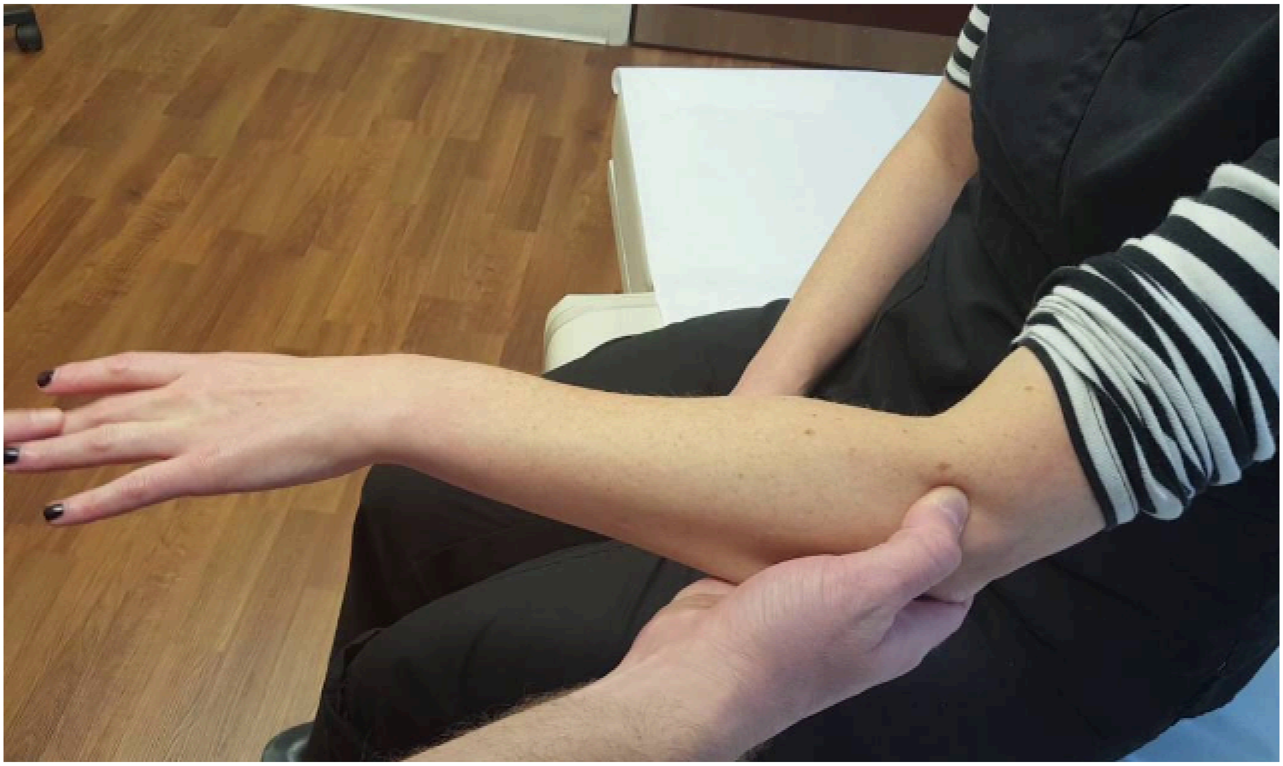

2. **Cozen’s test**: ایستاده، آرنج کاملا بحالت اکستانسیون و ساعد در پروناسیون ، شست درما اپی کندیل خارجی آرنج ، از بیمار می خواهیم با مشت کردن دست مچ دست را در برابر مقاومت ب سپس ساعد را به پروناسیون برده و بصورت پسیو مچ دست را به انحراف رادیال می بریم. درد آرنج نشانه مثبت بودن تست می‌باشد (30).

متن	عنوان
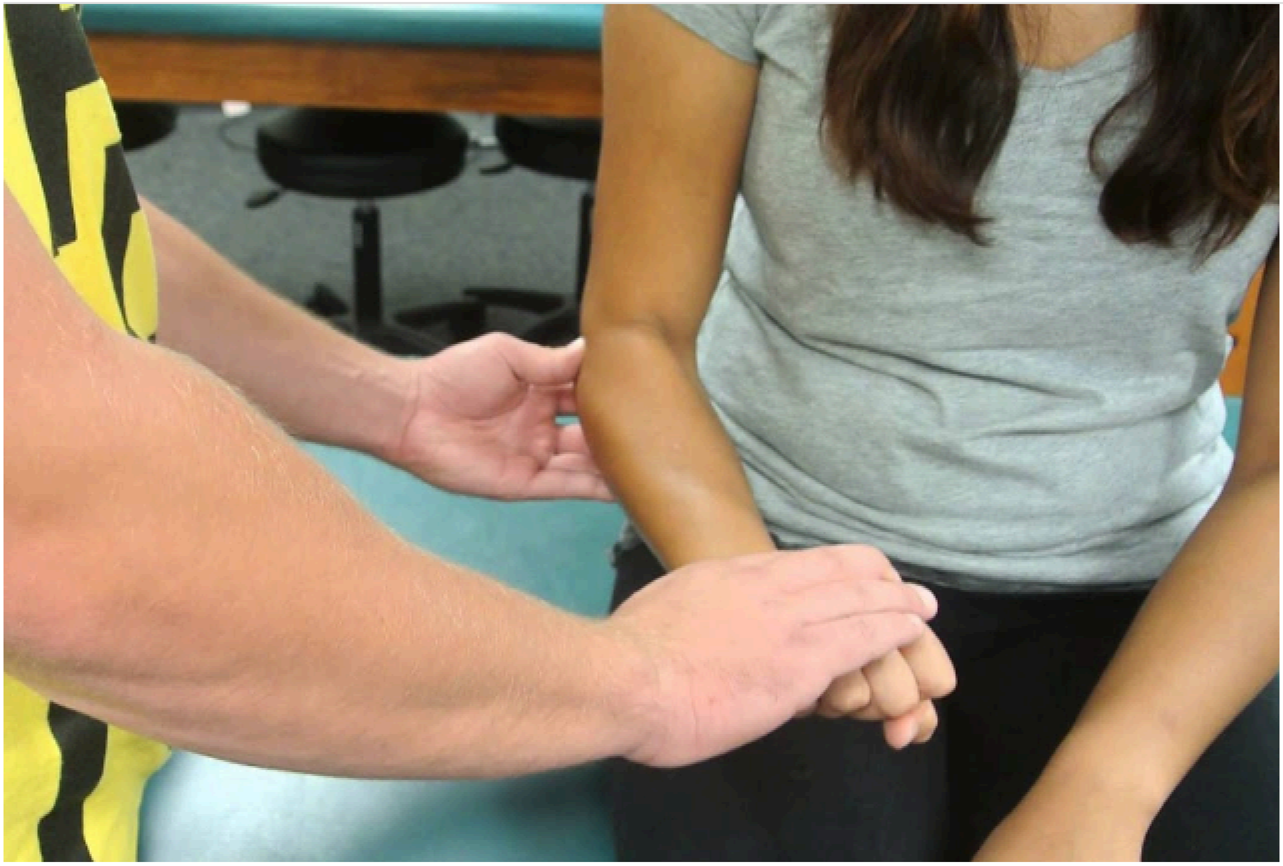<br><br>3. **Mill’s test:** در وضعیت نشسته، با وضعیت دهی شانه‌ها در دامنه ابتدایی حرکت ابداکسیون، فلکسیون، ساعد در پروناسیون و مچ دست در فلکسیون بطوریکه کف دست رو به سمت زمین باشد سمت مبتلا قرار گرفته و با یک دست بخش فوقانی بازو را نگه داشته و تا ۷۰ درجه ابداکسیون شست دست دیگر بر روی کف دست بیمار بین انگشت اشاره و شست قرار گرفته و انگشتان برای ا کامل ساعد در پشت مچ قرار می گیرند. در حالیکه این حالت را نگه می داریم به آهستگی اکس انجام می دهیم. درد در ناحیه خارجی آرنج نشانه این است که تست مثبت است (30).	

عنوان	متن

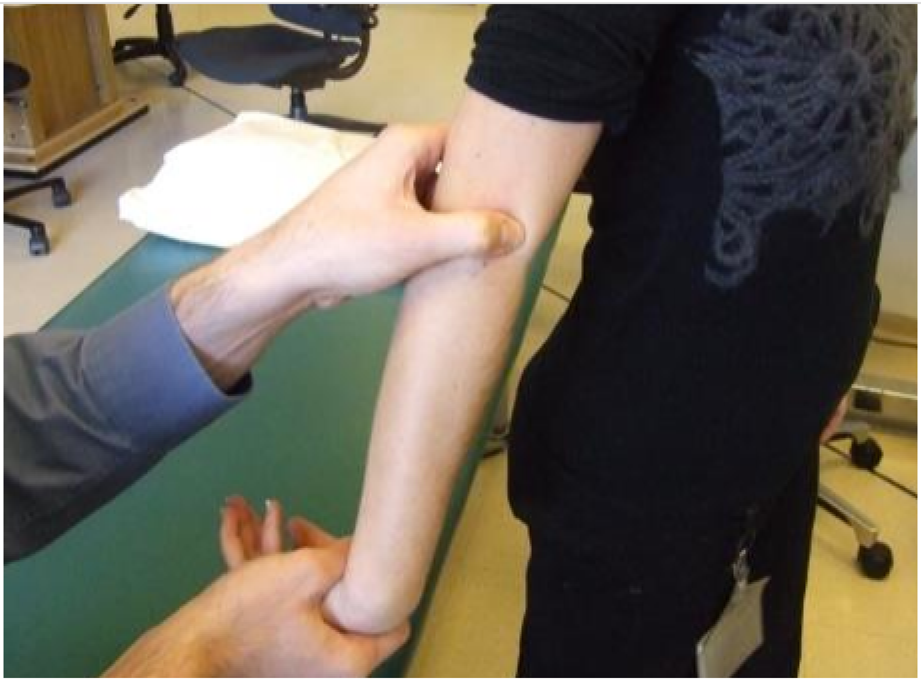

روش جمع آوری داده ها و نحوه اجرای مطالعه

خلاصه روش انجام کار:

 در مرحله اول این مطالعه، با هدف بررسی تکرارپذیری اندازه گیری های یک آزمونگر، اندازه گیری
اکستنسور مشترک در ناحیه اپی کندیل خارجی آرنج، با استفاده از دستگاه اولترا سونوگرافی رو
می شود و به مدت 48 ساعت بعد مجددا تکرار می شود.

این مطالعه به صورت کارآزمایی بالینی تصادفی و با طراحی parallel group designانجام خو
انتخاب بیماران بر اساس معیارهای ورود و خروج مطالعه، در تمامی افراد اطلاعات ographic
افراد، قد ، وزن، جنس، شغل، مدت درگیری، دست غالب، سمت درگیر و سابقه بیماری های زمینه ا
می شود. بیماران با درگیری یک طرفه وارد مطالعه خواهند شد و امتحان سونوگرافی از هردو
سالم بیمار در شروع مطالعه انجام می شود تا برآورد پایه از ضخامت تاندون مورد نظر در دو آرنج
به تمامی بیماران درباره مطالعه یک توضیح اجمالی داده می شود که هدف از انجام این مطالعه
اشخاص رضایت نامه کتبی آگاهانه گرفته می شود. گروه بندی در این مطالعه به صورت تصادفی
بیرونی در گروه بندی بیماران نقشی ندارند.

ارزیابی اولیه:

در ابتدای مطالعه، بررسی ضخامت تاندون اکستنسور مشترک، شدت درد کلی آرنج، قدرت گریپ بد
عملکردی اندام فوقانی در بیماران مبتلا به تنیس البو تحت ارزیابی قرار گرفته و ثبت خواهد شد.

عنوان	متن
	در گروه تحت درمان با کینزیوتیپ در ابتدا تکه کوچکی از کینزیوتیپ به ناحیه دیگری غیر از لترال سمت درگیر به مدت 24 ساعت چسبانده می شود تا از عدم حساسیت اطمینان حاصل شود (1 مطالعه، به همه بیماران آموزش داده خواهد شد که از فعالیت‌هایی مانند بلند کردن اجسام سنگین فشردن، ورز دادن خمیر، استفاده از ابزارهای کوچک دستی مانند مته یا پیچ گوشتی و باغبانی که بر روی تاندون عضلات اکستنسور مچ می شوند اجتناب کنند. ورزش خاصی برای هیچ یک از بیمه از گروه ها تجویز نمی شود. همچنین در طول مطالعه، بیماران مجاز به استفاده از داروهای خ نیستند (16).

مداخله:

گروه کانترفورس بریس:

از بیماران درخواست خواهد شد تا نقطه درد را بر روی آرنج نشان دهند و سپس به دنبال آن کانترف سانتی متر پایین تر از چین آرنج می بندیم. در ادامه از بیماران درخواست می شود تا حر اکستانسیون آرنج را به منظور اطمینان از عدم محدود شدن دامنه حرکتی آرنج به دنبال استفاد بریس، انجام دهند. همچنین از بیماران خواسته خواهد شد تا گریپ با نیروی زیاد انجام دهند تا مط زیاد سفت نمی شود (20, 28, 32). هر بیمار از کانترفورس بریس به مدت دو هفته استفاده خ بیمار فقط کانترفورس بریس را هنگام خواب و شستشو از آرنج در می آورد (21).از کانترفورس بازار در این مطالعه استفاده خواهد شد.

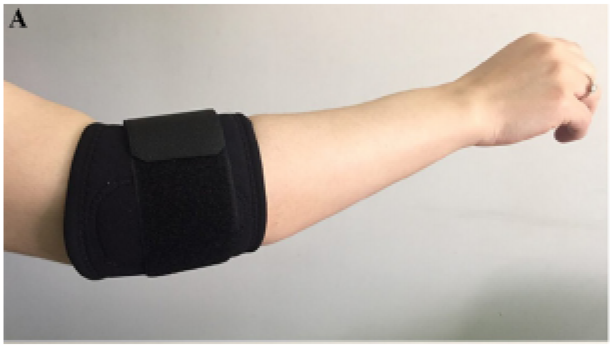

کانترفورس بریس برای بیماران تنیس البو

گروه کینزیوتیپ:

برای استفاده از کینزیوتیپ در جهت مهار عضله، از دو نوار Yشکل استفاده می کنیم. نوار اصل اکستنسور کارپی رادیالیس برویس و اکستنسور مشترک از قسمت دیستال عضله به سمت پروگزیم به منظور اصلاح فاسیا، با حداکثر کشش به صورت عمود بر نوار اصلی در قسمت فوقانی ساعد، چ وضعیت اندام فوقانی بیمار به صورت اکستانسیون آرنج و فلکسیون و انحراف مچ به سمت اول شود. قسمت ابتدا و انتهای تیپ اصلی باید بدون کشش متصل گردد. ولی در قرار دادن تیپ در ط کشش 30درصد چسبانده شود (32). تیپ ها هر3 روز به مدت دو هفته عوض می شوند. در مجمو

عنوان	متن
	کینزیوتیپ دریافت می کند (25).از بیماران حاضر در این گروه خواسته شد تا حد امکان ازتماس<br>آب جلوگیری شود (16). در این مطالعه، از کینزیوتیپ مارک bio balance tape استفاده خواهد [illegible]<br><br>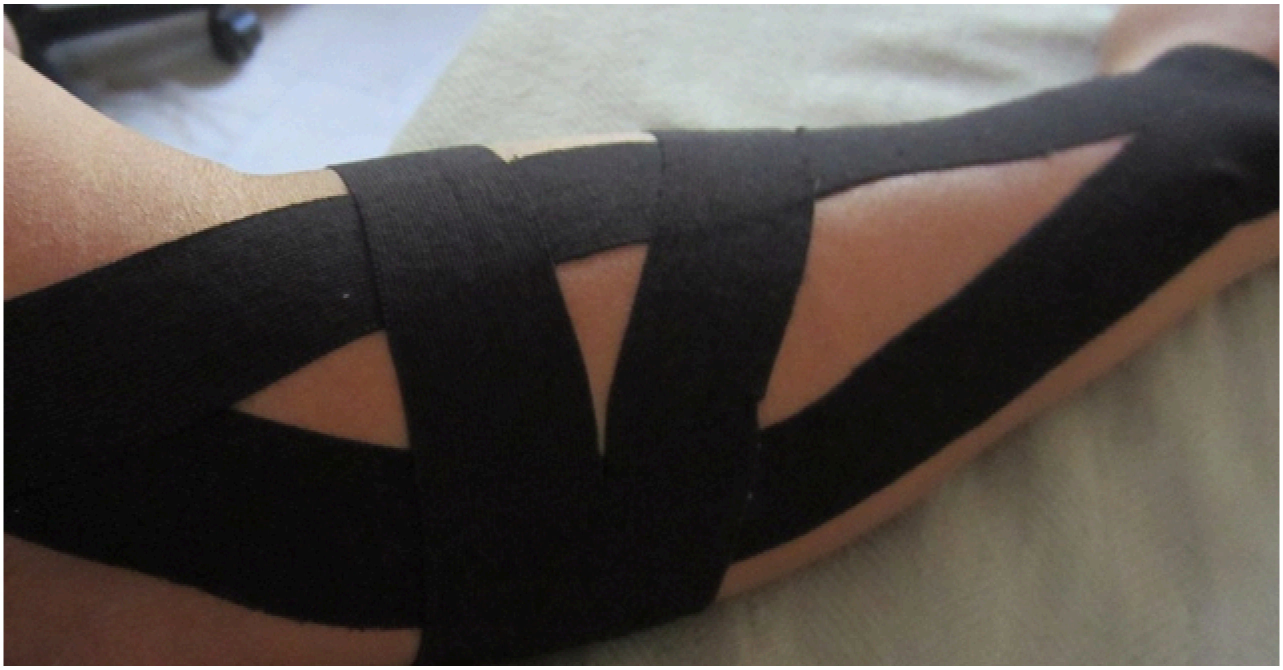<br><br>کینزیوتیپ برای بیماران تنیس البو<br><br>**گروه تزریق کورتیکو استرویید:**<br><br>در این گروه، بعد از ارزیابی اولیه، توسط پزشک ارتوپد به بیماران یک بار تزریق کورتیکو اس<br>لیدوکائین(یک میلی لیتر متیل پردنیزولون ده میلی گرمی به همراه یک میلی لیتر پری لوکائین دو<br>درد انجام می شود (15). آرنج بیمار در وضعیت 45درجه فلکسیون و پروناسیون ساعد بر روی یک<br>می گیرد (23).سوزن به صورت عمودی به محل درد وارد می شود تا به استخوان برخورد کند. سپ<br>که وارد بدن شد، خارج می شود و تزریق انجام می شود (27). هیچ روش مداخله ای دیگری اعم<br>داروهای خوراکی به بیماران تجویز نمی شود (15).
ابزارها	**پیامدهای[1] اولیه:**<br><br>***درد**<br><br>ارزیابی میزان تغییرات درد فرد مورد مطالعه با استفاده از پرسشنامه مقیاس دیداری درد (VAS)

متن	عنوان

***ضخامت تاندون اکستنسور مشترک**

اولتراسونوگرافی جهت اندازه گیری ضخامت تاندون عضله

بررسی سونوگرافی در دو گروه کینزیوتیپ و کانترفورس بریس ، ابتدا قبل از شروع درمان و پس درمانی و نهایتا دو هفته بعد از آخرین جلسه درمانی و در گروه تزریق کورتیکو استروئید، قبل از هفته و 4 هفته پس از انجام تزریق جهت بررسی پایایی اثر درمان توسط پزشک رادیولوژیست که بیماران بی اطلاع است، انجام می شود (33). در این بخش تغییرات ضخامت تاندون بررسی می حالت نشسته انجام شده به طوریکه آرنج در ۹۰ درجه فلکسیون و ساعد در حالت آنترومدیال و ا تخت معاینه قرار می‌گیرد (34). ضخامت تاندون عضلات اکستانسور براساس فاصله عمودی حدفاصل بین تاندون و سطح کورتکس اپی کندیل خارجی آرنج بیمار ارزیابی می شود (35). به مقطع قسمت وسط اپی کندیل هومروس، بالای سر رادیوس و بالای گردن رادیوس، ضخامت اکستنسور مشترک انگشتان اندازه گیری می شود (33). در این مطالعه، از دستگاه سونوگرافی Voluson E6 (با استفاده از پروب سطحی خطی و فرکانس15-12MHZ)ساخت کشور آمریکا شد.

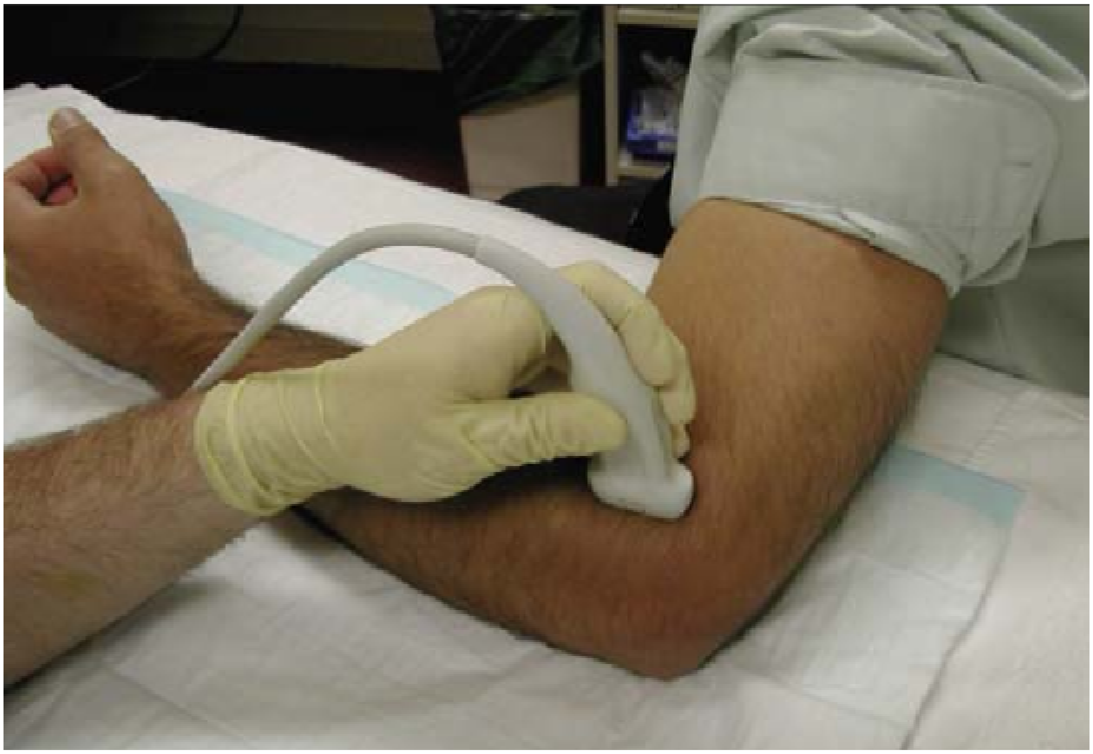

طریقه پوزیشن اندام فوقانی هنگام سونوگرافی آرنج در بیماران تنیس البو

عنوان	متن

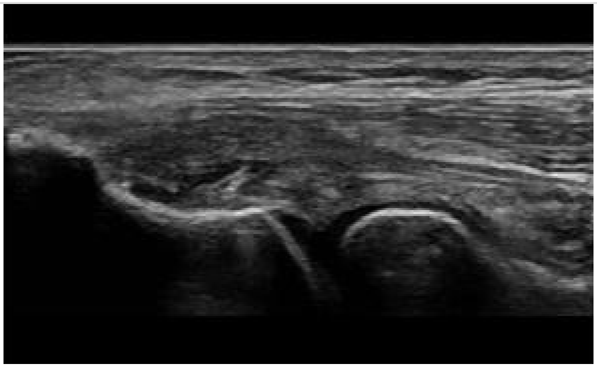

تصویر سونوگرافی ثبت شده از ضخامت تاندون در یک بیمار مبتلا به تنیس البو

پیامد های ثانویه :

***ناتوانی اندام فوقانی:**

با استفاده از پرسشنامه DASH که حاوی 30 سوال در مورد اختلال عملکرد ارتوپدیک و نورو فوقانی می باشد. هر سوال حاوی 5 گزینه که از 1 به معنی بدون سختی تا 5 به معنی عدم توانای فعالیت و شدیدترین علایم می باشد. امتیاز نهایی از 100 می باشد که 100 به معنی ناتوانی شدید نشانه درگیری کم تر می باشد. علاوه بر 30 سوال دو سری سوال با 4 آیتم وجود دارد که پاسخ به باشد که مشابه بالا امتیازبندی می شود (36). بر طبق این فرمول نمره به دست آمده از 30 کم می حداکثر نمره منهای حداقل نمره (30- 150) تقسیم بر 100 یعنی 2/1 می شود: (37)

[(Raw score - 30) ÷ (range of score)] ×100 =

***قدرت گریپ:**

اندازه گیری قدرت گریپ دست با استفاده از دینامومتر اندازه گیری می شود. برای انجام این تست مورد چگونگی طرز استفاده از این ابزار به بیمار داده می شود. بیمار بر روی صندلی با ارتفاع نشیند. شانه در وضعیت ابدکشن ۱۰ درجه و چرخش طبیعی، آرنج به حالت فلکشن ۹۰ درجه، د میانی و کنار بدن قرار می‌گیرد. سپس انقباضی با حداکثر قدرت و سرعت، جهت ثبت حداکثر ایزومتریک گرفته می شود، به طوریکه تغییرات در ضربان قلب و فشار خون ایجاد نشود. سپس خستگی بمدت ۱ دقیقه استراحت و سپس تست بعدی انجام می‌شود (در مجموع ۳ تست). بدین عددی ۳ تست ثبت می‌شود (38). از دستگاه دینامومتر ساخت شرکت Saehanmedical در این خواهد شد.

عنوان	متن
	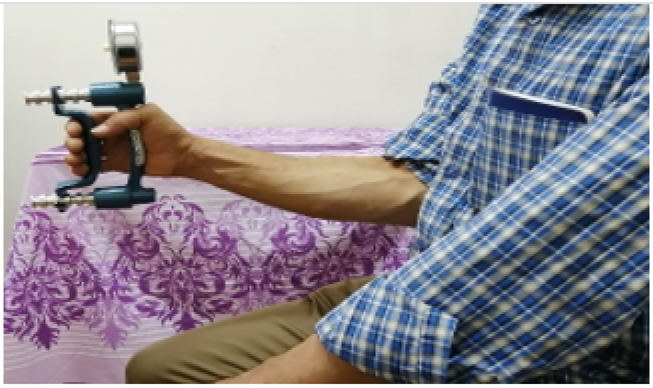<br>اندازه گیری قدرت گریپ با دستگاه دینامومتر<br>Outcome measures [1]
روش تجزیه تحلیل آماری	داده ها با استفاده از نرم افزار SPSS نسخه 22 مورد تجزیه و تحلیل قرار خواهند گرفت. آمار تو<br>از میانگین و انحراف معیار (برای دادهای کمی) و فراوانی و نسبت(برای داده های کیفی) ارایه خوا<br>منظور بررسی توزیع داده هااز آزمون های -SQUARE،T-TEST ndependent test<br>خواهد شد.بر حسب نتایج این آزمون از آزمون‌های پارامتری(nt sample<br>(test,paired sampels T-test, one way ANOVA ویا آزمون های غیرپاراما<br>(whitney U test, Wilcoxon singed rank test,chi-squared test اس<br>شد.سطح معنی داری هم 0.05 یا کمتر مساوی0.05 در نظر گرفته می شود

پیش بینی زمانبندی مراحل اجرای طرح

شرح مختصر مرحله -انتخابی	طول مرحله بر حسب ماه
تنظیم و تدوین طرح	2
مراحل اجرائي	6
تجزیه و تحلیل اطلاعات	1
گزارش نهائي طرح	3

کل مدت زمان : 0

ملاحظات اخلاقی

عنوان	توضیحات
ملاحظات اخلاقي	ازتمام افرادمورد آزمون درمطالعه موافقت آگاهانه اخذمي گردد.بیماران درهرمرحله ازدرمان وبه هردليلي مي توانندازمطالعة خارج شوند.مطالعه بعدازدريافت مجوزوکداخلاق ازدانشگاه علوم پزشکي بابل اجراخواهدشد.

هزینه پرسنلی

نوع پرسنل	مرتبه علمی	تعداد	میزان اشتغال - ساعت	حق الزحمه هر ساعت - ریال	کل حق الزحمه - ریال
مجریان	دانشجو	1	120	100,000	12,000,000
همکار/ مشاوره	استادیار	4	50	100,000	20,000,000
مجریان	دانشیار	1	100	100,000	10,000,000

جمع کل : 42,000,000

هزینه ازمایش و خدمات تخصصی

موضوع آزمایشات و یا خدمات تخصصی	مرکز سرویس دهنده	تعداد کل دفعات	هزینه برای هر دفعه	جمع کل هزینه ها
دستگاه اولتراسونوگرافي	بیمارستان شهید بهشتي - دانشكده توانبخشي	153	100000	15300000

جمع کل : 15,300,000

هزینه وسایل و مواد خریداری شده

نام دستگاه/ مواد	نوع وسیله	شرکت فروشنده /سازنده	کشور سازنده	موجود در ایران	تعداد	قیمت هر عدد به ریال	کل مبلغ
کانترفورس بریس	مصرفی		ایران	در بازار ایران موجود مي باشد	17	1400000	3800000
کینزیوتیپ	مصرفی	bio balance	کره	در بازار ایران موجود مي باشد	20	1500000	0000000
متیل پردنیزولون	مصرفی		ایران	در بازار ایران موجود مي باشد	17	100000	1700000
پری لوکائین	مصرفی		ایران	در بازار ایران	17	100000	1700000

نام دستگاه/ مواد	نوع وسیله	شرکت فروشنده /سازنده	کشور سازنده	موجود در ایران	تعداد	قیمت هر عدد به ریال	کل مبلغ
				موجود مي باشد			
سرنگ	مصرفی			در بازار ایران موجود مي باشد	17	50000	850000

جمع کل هزینه : 58,050,000

سایر هزینه ها

عنوان هزینه	مبلغ کل (ریال)
هزینه تکثیر اوراق	10000000
هزینه ارتباطات و حمل و نقل	3000000
سایر هزینه ها	2000000

جمع کل : 15,000,000

اعتبار مصوب برون سازمانی

برون‌سازمانی - پرسنلی	برون‌سازمانی - آزمایشگاه و خدمات تخصصی	برون‌سازمانی - مسافرت	برون‌سازمانی - متفرقه	برون‌سازمانی - وسایل و مواد غیر مصرفی	برون‌ساز[illegible] - وسایل [illegible] مواد مص[illegible]
رکوردي يافت نشد					

جمع هزینه های طرح

درخواست شده - پرسنلی	درخواست شده - آزمایشگاه و خدمات تخصصی	درخواست شده - وسایل و مواد خریداری شده	درخواست شده - متفرقه	کل اعتبار درخواست شده
42,000,000	15,300,000	58,050,000	15,000,000	130,350,000

فهرست منابع و ماخذ

منابع

1. Galván Ruiz A, Vergara Díaz G, Rendón Fernández B, Echevarría Ruiz De Vargas C. Effects of ultrasound-guided administration of botulinum toxin (incobotulinumtoxinA) in patients with lateral epicondylitis. Toxins. 2019;11(1):46.
2. Amro A, Diener I, Bdair WO, Isra'M H, Shalabi AI, Dua'l I. The effects of Mulligan mobilisation with movement and taping techniques on pain, grip strength, and function in patients with lateral epicondylitis. Hong Kong Physiotherapy Journal. 2010;28(1):19-23.
3. Amro A, Diener I, Isra'M H, Shalabi AI, Dua'l I. The effects of Mulligan mobilisation with movement and taping techniques on pain, grip strength, and function in patients with lateral epicondylitis. Hong Kong Physiotherapy Journal. 2010;28(1):19-23.
4. Nirschl RP, Pettrone F. Tennis elbow. The surgical treatment of lateral epicondylitis. The Journal of bone and joint surgery American volume. 1979;61(6A):832-9.
5. Kraushaar BS, Nirschl RP. Tendinosis of the elbow (tennis elbow): clinical features and findings of histological, immunohistochemical, and electron microscopy studies. Journal of Bone and Joint Surgery. 1999;81(2):259.
6. Coombes BK, Bisset L, Vicenzino B. A new integrative model of lateral epicondylalgia. British journal of sports medicine. 2009;43(4):252-8.
7. Manickaraj N, Bisset LM, Kavanagh JJ. Lateral epicondylalgia exhibits adaptive muscle activation strategies based on wrist posture and levels of grip force: a case-control study. Journal of musculoskeletal & neuronal interactions. 2018;18(3):323.
8. Coombes BK, Bisset L, Vicenzino B. Management of Lateral Elbow Tendinopathy: One Size Does Not Fit All. The Journal of orthopaedic and sports physical therapy. 2015;45(11):938-49.
9. Ahmad Z, Siddiqui N, Malik S, Abdus-Samee M, Tytherleigh-Strong G, Rushton N. Lateral

epicondylitis: a review of pathology and management. The bone & joint journal. 2013;95(9):1158-64.

10. Kotnis NA, Chiavaras MM, Harish S. Lateral epicondylitis and beyond: imaging of lateral elbow pain with clinical-radiologic correlation. Skeletal radiology. 2012;41(4):369-86.

11. Ma K-L, Wang H-Q. Management of Lateral Epicondylitis: A Narrative Literature Review. Pain Research and Management. 2020;2020.

12. Uygur E, Aktaş B, Özkut A, Erinc S, Yilmazoglu EG. Dry needling in lateral epicondylitis: a prospective controlled study. International orthopaedics. 2017;41(11):2321-5.

13. Ozkut AT, Kilinçoğlu V, Ozkan NK, Eren A, Ertaş M. Extracorporeal shock wave therapy in patients with lateral epicondylitis. Acta Orthop Traumatol Turc. 2007;41(3):207-10.

14. Tsikopoulos K, Tsikopoulos I, Simeonidis E, Papathanasiou E, Haidich A-B, Anastasopoulos N, et al. The clinical impact of platelet-rich plasma on tendinopathy compared to placebo or dry needling injections: A meta-analysis. Physical Therapy in Sport. 2016;17:87-94.

15. Arik HO, Kose O, Guler F, Deniz G, Egerci OF, Ucar M. Injection of autologous blood versus corticosteroid for lateral epicondylitis: a randomised controlled study. Journal of Orthopaedic Surgery. 2014;22(3):333-7.

16. Erpala F, Ozturk T, Zengin EC, Bakir U. Early Results of Kinesio Taping and Steroid Injections in Elbow Lateral Epicondylitis: A Randomized, Controlled Study. Medicina. 2021;57(4):306.

17. Stasinopoulos D, Stasinopoulos I. Comparison of effects of eccentric training, eccentric-concentric training, and eccentric-concentric training combined with isometric contraction in the treatment of lateral elbow tendinopathy. Journal of hand therapy. 2017;30(1):13-9.

18. Bisset LM, Collins NJ, Offord SS. Immediate effects of 2 types of braces on pain and grip strength in people with lateral epicondylalgia: a randomized controlled trial. journal of orthopaedic & sports physical therapy. 2014;44(2):120-8.

19. Au IP, Fan PCP, Lee WY, Leong MW, Tang OY, An WW, et al. Effects of Kinesio tape in individuals with lateral epicondylitis: a deceptive crossover trial. Physiotherapy theory and practice. 2017;33(12):914-9.

20. Barati H, Zarezadeh A, MacDermid JC, Sadeghi-Demneh E. The immediate sensorimotor effects of elbow orthoses in patients with lateral elbow tendinopathy: a prospective crossover study. Journal of shoulder and elbow surgery. 2019;28(1):e10-e7.

21. Dundar U, Turkmen U, Toktas H, Ulasli AM, Solak O. Effectiveness of high-intensity laser therapy and splinting in lateral epicondylitis; a prospective, randomized, controlled study. Lasers in medical science. 2015;30(3):1097-107.

22. Öken Ö, Kahraman Y, Ayhan F, Canpolat S, Yorgancioglu ZR, Öken ÖF. The short-term efficacy of laser, brace, and ultrasound treatment in lateral epicondylitis: a prospective, randomized, controlled trial. Journal of Hand Therapy. 2008;21(1):63-8.

23. Koçak FA, Kurt EE, Sas S, Tuncay F, Erdem HR. Short-term effects of steroid injection, kinesio taping, or both on pain, grip strength, and functionality of patients with lateral epicondylitis: A single-blinded randomized controlled trial. American journal of physical medicine & rehabilitation. 2019;98(9):751-8.

24. Özmen T, Koparal SS, Karataş Ö, Eser F, Özkurt B, Gafuroğlu TÜ. Comparison of the clinical and sonographic effects of ultrasound therapy, extracorporeal shock wave therapy, and Kinesio taping in lateral epicondylitis. Turkish Journal of Medical Sciences. 2021;51(1):76-83.

Giray E, Karali-Bingul D, Akyuz G. The effectiveness of Kinesiotaping, sham taping or .25
exercises only in lateral epicondylitis treatment: a randomized controlled study. Pm&r.
.2019;11(7):681-93

Eraslan L, Yuce D, Erbilici A, Baltaci G. Does Kinesiotaping improve pain and functionality .26
in patients with newly diagnosed lateral epicondylitis? Knee Surg Sports Traumatol Arthrosc.
.2018;26(3):938-45

Hsieh L-F, Kuo Y-C, Lee C-C, Liu Y-F, Liu Y-C, Huang V. Comparison between corticosteroid .27
and lidocaine injection in the treatment of tennis elbow: a randomized, double-blinded,
.controlled trial. American journal of physical medicine & rehabilitation. 2018;97(2):83-9

Kroslak M, Pirapakaran K, Murrell GA. Counterforce bracing of lateral epicondylitis: a .28
prospective, randomized, double-blinded, placebo-controlled clinical trial. Journal of shoulder
.and elbow surgery. 2019;28(2):288-95

George CE, Heales LJ, Stanton R, Wintour S-A, Kean CO. Sticking to the facts: a .29
systematic review of the effects of therapeutic tape in lateral epicondylalgia. Physical
.Therapy in Sport. 2019;40:117-27

Saroja G, Aseer P, Venkata Sai P. Diagnostic accuracy of provocative tests in lateral .30
.epicondylitis. Int J Physiother Res. 2014;2(6):815-23

Kase K. Clinical therapeutic applications of the Kinesio (! R) taping method. Albuquerque. .31
.2003

Cho Y-T, Hsu W-Y, Lin L-F, Lin Y-N. Kinesio taping reduces elbow pain during resisted wrist .32
extension in patients with chronic lateral epicondylitis: a randomized, double-blinded, cross-
.over study. BMC musculoskeletal disorders. 2018;19(1):1-8

Samiei SM, Aminian Far A, Paknazar F. Ultra-sonographic study of the effect of dry .33
needling of extensor muscles combined with Mulligan mobilization technique on extensor
muscle tendon thickness, pain and upper limb function in patients with lateral epicondylitis: A
.double-blinded randomized clinical trial. Koomesh journal. 2021;23(6):730-40

Clarke AW, Ahmad M, Curtis M, Connell DA. Lateral elbow tendinopathy: correlation of .34
ultrasound findings with pain and functional disability. The American journal of sports
.medicine. 2010;38(6):1209-14

Mohan PC, editor Lateral elbow tendinopathy: correlation between ultrasound findings .35
and clinical outcomes after percutaneous ultrasonic tenotomy2015: European Congress of
.Radiology 2015

MAHMOUDI HF, Mousavi J, ATTARBASHI MB, TALEBIAN MS, Mousavi S. Cross cultural .36
adaptation, Validity and Reliability study of the Persian version of the American shoulder and
elbow surgeons (ASES) Questionnaire for evaluation of shoulder function in injured athletes.
.2013

Mohseni Bandpei MA, Keshavarz R, Minoonejad H, Ebrahimi Varkiani M, Samadi H, Latifi .37
S. Shoulder pain and functional disability in Iranian premier league volleyball players. Journal
.of Mazandaran University of Medical Sciences. 2012;22(90):95-103

Kuzala EA, Vargo MC. The relationship between elbow position and grip strength. .38
.American Journal of Occupational Therapy. 1992;46(6):509-12
